# Revisions to Chronic Disease Surveillance Indicators, United States, 2004

**Published:** 2005-06-15

**Authors:** Andrew R Pelletier, Paul Z Siegel, Mark S Baptiste, Christopher Maylahn

**Affiliations:** Maine Bureau of Health; Dr Pelletier is also affiliated with the Centers for Disease Control and Prevention, Atlanta, Ga; Centers for Disease Control and Prevention, Atlanta, Ga; New York State Department of Health, Albany, NY, and Council of State and Territorial Epidemiologists, Atlanta, Ga; New York State Department of Health, Albany, NY, and Association of State and Territorial Chronic Disease Program Directors, McLean, Va

## Abstract

To allow public health officials to uniformly define, collect, and report chronic disease data, *Indicators for Chronic Disease Surveillance* was released by the Council of State and Territorial Epidemiologists in 1999. This publication provided standard definitions for 73 indicators developed by epidemiologists and chronic disease program directors at the state and federal levels. The indicators were selected because of their importance to public health and the availability of state-level data. This report describes the latest revisions to the chronic disease indicators published in 2004. The revised set of 92 indicators includes 24 for cancer; 15 for cardiovascular disease; 11 for diabetes; 7 for alcohol; 5 each for nutrition and tobacco; 3 each for oral health, physical activity, and renal disease; and 2 each for asthma, osteoporosis, and immunizations. The remaining 10 indicators cover such overarching conditions as poverty, education, and life expectancy. Although multiple states have used the indicators, wider adoption depends on increased epidemiology capacity at the state level and improved access to surveillance data.

## Chronic Disease Surveillance Indicators

In 1999, the Council of State and Territorial Epidemiologists (CSTE) released *Indicators for Chronic Disease Surveillance* (1), a publication that provided standard definitions for 73 indicators developed by epidemiologists and chronic disease program directors at the state and federal levels. The indicators were selected because of their importance to public health and the availability of state-level data and were intended to allow states and territories to uniformly define, collect, and report chronic disease data. In 2000, CSTE released a companion volume that included the most current data for the indicators for each state, the District of Columbia, and Puerto Rico (2). This report describes the revised chronic disease indicators and data sources for the indicators published in 2004 (3).

## Revision of the Chronic Disease Indicators

Revision of the original 73 indicators began in 2000 with the formation of a work group composed of representatives of CSTE, the Association of State and Territorial Chronic Disease Program Directors, and the National Center for Chronic Disease Prevention and Health Promotion of the Centers for Disease Control and Prevention (CDC). The chronic disease indicators were developed to be consistent with the national health objectives of *Healthy People 2010* (4), whenever state-level data were available for chronic disease objectives. A draft set of indicators was distributed to all state health departments for comment. After further revisions, 36 national health organizations were asked to review the indicators. The new set of 92 indicators was approved at the annual CSTE meeting in 2002 and is available from www.cdc.gov/nccdphp/cdi. State-specific data will be available at this site at a later date.

## Categories and Data Sources

The indicators are divided into six categories: cancer, cardiovascular disease, tobacco and alcohol use, physical activity and nutrition, other diseases and risk factors, and overarching conditions. Sixty-three (68%) of the 92 indicators are unchanged from the first edition, 6 (7%) were revised, and 23 (25%) are new. Four indicators from the first edition were deleted. Of the indicators, 24 (26%) are for cancer; 15 (16%) for cardiovascular disease; 11 (12%) for diabetes; 7 (8%) for alcohol; 5 (5%) each for nutrition and tobacco; 3 (3%) each for oral health, physical activity, and renal disease; and 2 (2%) each for asthma, osteoporosis, and immunizations. The remaining 10 (11%) indicators cover overarching conditions (e.g., poverty, education, life expectancy, health insurance).

Data for the indicators were derived from nine sources. Data for 34 (37%) indicators are from the Behavioral Risk Factor Surveillance System (BRFSS), 24 (26%) from vital statistics, 12 (13%) from hospital discharge data, 9 (10%) from cancer registries, 6 (7%) from the Youth Risk Behavior Surveillance System (YRBSS), 2 (2%) from either the YRBSS or the Youth Tobacco Survey (YTS), 2 (2%) from the United States Renal Data System (USRDS), 2 (2%) from the Current Population Survey (CPS), and 1 (1%) from state revenue departments.

All states, the District of Columbia, and Puerto Rico report annual data from the BRFSS, vital statistics, the USRDS, and state revenue departments, and all have cancer registries (Table). A total of 38 (76%) states and the District of Columbia were certified by the North American Association of Central Cancer Registries (NAACCR) for 2001 incidence data (5). The CPS includes all states and the District of Columbia but not Puerto Rico. As of 2004, a total of 46 (92%) states and the District of Columbia had hospital discharge data systems (Agency for Healthcare Research and Quality, unpublished data, 2004). In 2003, 32 (64%) states and the District of Columbia participated in the YRBSS and produced weighted data (6). During 2002–2003, a total of 43 (86%) states, the District of Columbia, and Puerto Rico participated in the YRBSS (6) or YTS (CDC, unpublished data, 2004) and produced weighted data.

## Uses of the Chronic Disease Indicators

Chronic diseases account for 7 of the 10 leading causes of death in the United States, including diseases associated with the three leading causes of preventable death: tobacco use, improper diet and physical inactivity, and alcohol use (7,8). Approximately 70% of health-care costs in the United States are for chronic diseases (7). Public health surveillance is necessary to monitor progress in controlling chronic diseases.

States have used the chronic disease indicators in a variety of ways. Georgia calculated values for most of the indicators for its 19 health districts and plans to create a database with standardized reports for each health district and post the data on the Internet. New Mexico published a comprehensive chronic disease surveillance report that examined the available data for each indicator. Whenever possible, data were presented at the district and county levels (9). New Hampshire used the indicators to develop the state's diabetes surveillance system; 12 of the 13 measures in the state's surveillance system were from the chronic disease indicators (10). In Ohio, the indicators helped to improve program evaluation by ensuring that epidemiological data were used systematically for baseline measurements in program impact and outcome objectives. Oregon used the indicators to standardize analysis of chronic disease surveillance data. These data helped to guide chronic disease prevention efforts, including activities aimed at reducing health disparities (11). Maine used the indicators for guidance in developing county-level fact sheets on cardiovascular disease (12).

At the federal level, the Division of Diabetes Translation at the CDC used the chronic disease indicators as a model to develop the Diabetes Indicators and Data Sources Internet Tool (DIDIT). This tool contains 38 diabetes indicators and lists associated national and state data sources. DIDIT is designed to assist diabetes programs with surveillance and epidemiologic activities (13). (For more information on the DIDIT, see Mukhtar et al in this issue of *Preventing Chronic Disease* [14]). In addition, the National Oral Health Surveillance System (NOHSS) was developed based on the framework for the chronic disease indicators (15). Three of the eight measures in NOHSS are currently included in the chronic disease indicators.

There are at least two limitations to wider use of the chronic disease indicators. First, not all data sources are universally available. Only 22 (44%) of the states and the District of Columbia have access to the recommended data from all nine of the data sources used for the chronic disease indicators. Second, not all states have sufficient chronic disease epidemiology capacity to collect, analyze, and report on the data required for each indicator. According to a 2004 survey by the CSTE, 43% of responding states did not have a state chronic disease epidemiologist (16).

The chronic disease indicators facilitate and standardize surveillance at both the state and national levels. The indicators should be reviewed periodically because of changes in availability of data and public health priorities for chronic disease. Expanding the use of the chronic disease indicators will depend upon enhanced chronic disease epidemiology capacity at the state level and improved access to surveillance data.

## Figures and Tables

**Table:** Available Data Sources for Chronic Disease Indicators, by State or Area, United States, 2004^a^

**State**	**Cancer Registry (2001)**	**Hospital Discharge Data**	**YRBSS^b^ (2003)**	**YTS^b^(2002-2003) **
Alabama	X		X	X
Alaska	X	X	X	
Arizona	X	X	X	X
Arkansas	X	X	X^c^	X^c^
California	X^d^	X		X
Colorado	X	X	X^c^	X
Connecticut	X^d^	X	X^c^	X
Delaware	X^d^	X	X	X
District of Columbia	X	X	X	
Florida	X	X	X	X
Georgia	X	X	X	
Hawaii	X	X	X^c^	X
Idaho	X		X	
Illinois	X	X		X
Indiana	X	X	X	X
Iowa	X	X	X^c^	X
Kansas	X	X	X^c^	X
Kentucky	X	X	X	X
Louisiana	X	X	X^c^	
Maine	X	X	X	
Maryland	X^d^	X		X
Massachusetts	X	X	X	X
Michigan	X	X	X	
Minnesota	X	X		X^c^
Mississippi	X^d^		X	X
Missouri	X	X	X	X
Montana	X		X	
Nebraska	X	X	X	X
Nevada	X	X	X	
New Hampshire	X	X	X	
New Jersey	X	X	X^c^	
New Mexico	X	X	X^c^	X
New York	X	X	X	X
North Carolina	X	X	X	X
North Dakota	X^d^	X	X	X
Ohio	X	X	X	X
Oklahoma	X	X	X	X
Oregon	X	X	X^c^	
Pennsylvania	X^d^	X		X
Puerto Rico	X^d^			X
Rhode Island	X	X	X	X
South Carolina	X	X	X^c^	
South Dakota	X^d^	X	X	X
Tennessee	X^d^	X	X	X
Texas	X^d^	X	X	
Utah	X	X	X	X
Vermont	X^d^	X	X	
Virginia	X^d^	X		X
Washington	X	X		
West Virginia	X	X	X	X
Wisconsin	X	X	X	X
Wyoming	X	X	X	

a X denotes that the data source is available; no entry indicates that the data source is not available. All U.S. states, the District of Columbia, and Puerto Rico report annual data from the Behavioral Risk Factor Surveillance System, U.S. Renal Data System, state revenue departments, and vital statistics. All states and the District of Columbia report data from the Current Population Survey.

b YRBSS indicates Youth Risk Behavior Surveillance System; YTS, Youth Tobacco Survey

c Unweighted data.

d States that were not certified by the North American Association of Central Cancer Registries for 2001.
